# Comparative transcriptome analysis of translucent flesh disorder in mangosteen (*Garcinia mangostana L.*) fruits in response to different water regimes

**DOI:** 10.1371/journal.pone.0219976

**Published:** 2019-07-19

**Authors:** Deden Derajat Matra, Toshinori Kozaki, Kazuo Ishii, Roedhy Poerwanto, Eiichi Inoue

**Affiliations:** 1 Department of Agronomy and Horticulture, IPB University (Bogor Agricultural University), Bogor, West Java, Indonesia; 2 College of Agriculture, Ibaraki University, Ami, Ibaraki, Japan; 3 Biostatistics Center, Kurume University, Kurume, Fukuoka, Japan; University of Western Sydney, AUSTRALIA

## Abstract

Translucent flash disorder (TFD) is one of the important physiological disorders in mangosteen (*Garcinia mangostana* L.). TFD has symptoms such as flesh arils that become firm and appear transparent similar to watercore in apple or pear. Information on the changes of gene expression in TFD-affected tissues remain limited, and investigations into the effects of different water regimes still need to be undertaken. Through an RNA sequencing approach using the Ion Proton, 183,274 contigs with length ranging from 173–13,035 bp were constructed by de novo assembly. Functional annotation was analyzed using various public databases such as non-redundant protein NCBI, SwissProt, and Gene Ontology, and KEGG pathway. Our studies compared different water regimes to incidence and differentially expressed genes of TFD-like physiological disorders. From the differentially expressed gene (DEG) between normal air and TFD-affected aril, we identified DEG-related TFD events, which 6228 DEGs in the control condition and 3327 DEGs in under water stress treatment condition remained, and confirmed these with RT-qPCR, including sucrose synthase (SUSY), endoglucanase (GUN), xyloglucan endotransglucosylase/hydrolase (XTH), and polygalacturonase (PG) showed statistically significant. In addition, transcription factors also indicated changes in MYB, NAC and WRKY between tissues and different water regimes.

## Introduction

Plant physiological disorders occur during plant development; these are not visible and are usually difficult to identify from outside [1]. In horticultural products such as fruits that are consumed as a fresh product, the appearance of physiological disorders influences consumer tastes, downgrades quality, and lowers pricing. The postharvest losses in fresh-consumed horticultural products are estimated at approximately 5–35% in developed countries, and this increases to approximately 20–50% in developing countries [2]. One of the quality losses in fruits are caused by the appearance of physiological disorders, especially in tropical fruit crops such as mangosteen (translucent flesh disorder, TFD), citrus (cracking and puffing), mango (spongy tissue and internal breakdown), guava (bronzing), and pomegranate (fruit cracking and aril browning) [3,4].

Mangosteen (*Garcinia mangostana* L.) is a tropical fruit with high economical, nutritional, and health values. In Indonesia, most provinces produce mangosteen fruits, with total production increasing from 117,595 tons in 2011 to 203,100 tons in 2015. In total, 98,294.6 tons were exported in 2015, with a market value of US$ 44.9 million, and were exported to Asian, Middle East, and some European countries [5]. However, fruits with physiological disorders such as TFD are generally rejected by consumers less than 20% of total production.

TFD is one of the major physiological disorders in mangosteen. TFD symptoms cause the flesh to appear transparent or water soaked, and in severe cases, may become firm and crisp. Disorders with symptoms similar to TFD in mangosteen have been reported and differentially termed depending on the fruit, such as watercore in apple [6], European pear [7], and Japanese pear [8]; water-soaked brown flesh disorder [9]; water-soaking in melon [10]; and flesh translucency in pineapple [11]. The phenomenon is not confined to fruits, similar symptoms have also been found in onion and termed translucent scale (Shock et al., 2007) [12]. In previous studies, specific gravity techniques have been used to separate TFD-affected from healthy mangosteen fruits [3]. Recently, more reliable and accurate techniques such as near infrared reflectance spectroscopy [13], vibration frequency base on strain gage sensor [14], electrical resistance and capacitance [15], and electrical impedance [16], have been used to predict TFD-affected fruits.

The causes of TFD are still unclear and the subject of speculation. However, mechanical injuries during harvesting and handling [17], water-related stress [18,19], and nutrient deficiency [20,21] are highly correlated to occurrences of TFD in mangosteen. The incidence of TFD increased when there was excess water during fruit development, around 9 weeks after anthesis, and when plants were re-watered suddenly after drying conditions to -100 kPa soil moisture, which trigger the increase in incidence of TFD-affected mangosteen fruits [19,22]. The intensive irrigation increased TFD-affected fruits [23]. Calcium and boron concentrations in TFD-affected flesh were different compared to normal flesh, in which calcium was lower and boron was higher [20]. Application of CaCl_2_ and H_3_BO_4_ by spraying decreased the percentage of TFD-affected fruits [21]. Some studies also suggest that changes in the cell wall led to the occurrence of TFD. Changes in the ultrastructure and biochemistry of the cell wall in TFD-affected flesh have been observed. TFD-affected cell walls showed that the cells had loss disintegration between middle lamella and primary cell wall and that they were swollen [24]. Pectin composition, as well as calcium distribution on the pectin, was higher in TFD than normal flesh [25].

Recently, massively parallel sequencing technologies such as next generation sequencing (NGS), have provided immense opportunities to obtain massive amounts of sequence data at very low cost and with high time efficiency compared to Sanger sequencing. Using NGS platforms allows the sequencing of all transcripts concurrently and can also quantify the gene expression level [26]. Although there have been various studies on TFD-like physiological disorders in mangosteen at the physiological and morphological levels, molecular level details have not been reported yet. Here, we used NGS platforms based on Ion Proton systems for RNA sequencing to obtain the reliable transcriptome reference for mangosteen and analysis of their differential gene expression in instances of translucent flesh disorder in mangosteen. Our studies compare different water regimes to incidence and differentially expressed genes of TFD-like physiological disorders. In further studies, these results can be used for better understanding of gene regulation and mechanisms of TFD-like physiological disorders in mangosteen.

## Materials and methods

### Plant materials

Fruits were collected from mature mangosteen trees, which were grown and maintained at the Pasir kuda experimental field, Center for Tropical Horticultural Studies, Bogor Agricultural University (Bogor, Indonesia). The experimental conditions used four treatments in which started 7 weeks anthesis, including (1) control as the natural growth condition (Control), (2) irrigating the land surface under the canopy (about 50 cm from trunk) with drip irrigation for the whole day (ILS), (3) covering the land surface under the canopy with dark plastic sheet (CLS), and (4) the treatments condition copied from 3rd treatment (CLS condition), with applied water irrigation every morning (CLS+RW). Twenty fruits were collected from each treatment randomly, then the incidences of translucent flesh disorder (TFD) was characterized by scoring each fruit on a scale of 0–4 [27]. A score of 0 (none) was assigned when no part of the fruit was affected, and 1 (slight), 2 (moderate), 3 (severe), and 4 (most severe) were assigned when less than 25%, 50%, 75%, and 100% of the fruit was affected, respectively. The data were analyzed using SAS University Edition. Analysis of variance (ANOVA) was used to test for significant differences between treatments, and Duncan’s Multiple Range Test was used to compare means within the ANOVA.

**Fig 1:**
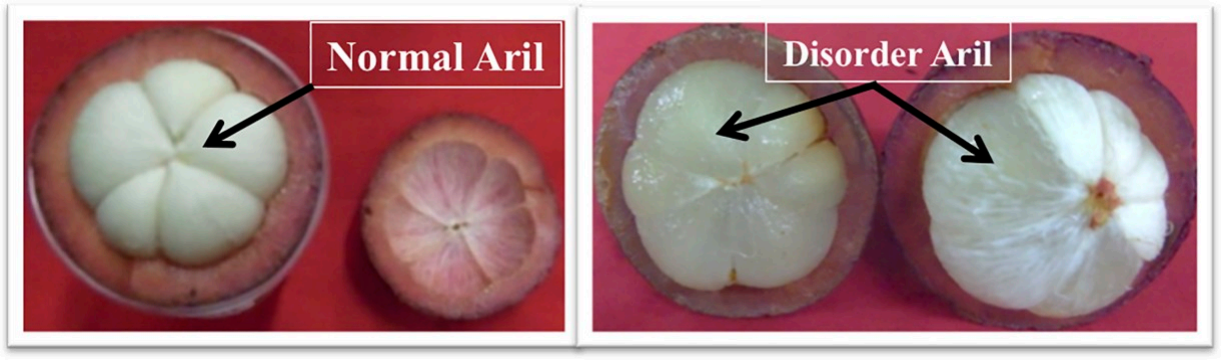
**Appearances of translucent flesh disorder-affected aril of (right and center) and normal aril (left) in mangosteen fruit.** This photo was taken by DDM.

For RNA extraction, the fruit was harvested and collected from a normal aril and translucent affected-aril with three replicates per treatment. All samples were stored in RNALater solution (Life Technologies, Grand Island, NY) prior to RNA extraction at 4°C. For RNA sequencing, 12 libraries were constructed from normal and TFD-affected arils of both of control and CLS treatments. The RNA sample were collected from two tissue type including normal aril and TFD-affected aril (Fig 1)

### RNA isolation, sequencing, data pre-processing, de novo transcriptome assembly, and contigs construction

Total RNA was extracted using the hot-borate method [28]. Quantity and quality of total RNA were measured using the NanoDrop ND-1000 spectrophotometer (Thermo Scientific, Wilmington, DE) and the 2100 Bioanalyzer (Agilent, Santa Clara, CA), respectively. PolyA RNA was subsequently isolated from 1 μg total RNA using the Dynabeads mRNA DIRECT Micro Purification Kit (Life Technologies, Grand Island, NY). The 12 libraries were constructed using the Ion Total RNA-Seq Kit v2 and Ion PI Template OT2 200 Kit v3 (Life Technologies, Grand Island, NY) following the manufacturer’s protocols. RNA sequencing was performed using the Ion PI Sequencing 200 kit v3 on the Ion Proton System (Life Technologies, Grand Island, NY).

To perform transcriptome assembly, the raw data was obtained from DRA005014 [29], DRA005254, and DRA005255 (in this study). Data pre-processing was performed following [29] to obtain clean data without rDNA filtering. De novo assembly of the transcriptome was performed by Trinity 2.2.0 [30] with default parameters to set a minimum length of 200 bp and performing an *in silico* normalization to set coverage at 30x. To reduce redundant contigs, the contigs were performed using the CAP3 program with parameters, -p 90 [31] and CD-HIT-EST with parameters, -c 0.90 -M 0 -T 0 [32]. The ribosomal DNA (rDNA) sequences were removed from the contigs using the BLAST+ program [33] with megablast parameter matched to the SILVA rRNA 128 databases [34] and the mangosteen rDNA sequences downloaded from NCBI database. Then, rRNA-removed contigs were hierarchically clustered using the Corset 1.06 program [35] as the mangosteen reference transcriptome and then deposited as a transcriptome shotgun assembly (TSA) to DDBJ with accession numbers, IACD01000001-IACD01183274. The high-quality reads were mapped to the rRNA-removed contigs using the SortMeRNA program with default parameters [36] to remove potential rDNA from reads.

### Functional annotation, classifications of contigs, and differential gene expression (DGE) analysis

The contigs were aligned using the BLAST+ program against the NCBI non-redundant databases (nr/nt) with gi accession subset to Viridiplantae and UniProtKB databases (Swiss-Prot and TrEMBL subset to Viridiplantae databases) with an E-value cut-off of 10^−5^. Gene Ontology (GO), Kyoto Encyclopedia of Genes and Genomes (KEGG), and INTERPRO were performed using BLAST2GO software [37] retrieved from BLASTX-contigs against the NCBI protein non-redundant database. To identify putative transcription factors (TFs), transcriptional regulators (TRs), and protein kinases (PKs), the contigs were performed using the iTAK program [38].

To determine the differential expressed genes, high quality reads from each sample were mapped onto reference transcriptome sequences using Bowtie2 with default parameters [39]. The abundance of each transcript was calculated using eXpress software [40]. The R package edgeR [41] was used to identify differential expression of transcripts across samples with three biological replicates and directly used for comparing among tissue types, namely normal and TFD tissues, and conditions, namely control and treatment respectively. The significance of differential expression was considered by the fold change (>2) and adjusted-p value using Benjamini and Hochberg’s approach [42] approach to obtain the false discovery rate (FDR > 0.01). Gene ontology of DEGs from each pairwise comparison was performed using the DAVID 6.8 beta web interface [43,44]. KEGG analysis of DEGs from each pairwise comparison was performed using the KOBAS 2.0 web interface [45].

Twenty-one genes were selected from contigs to validate their reliability and actin gene (C12506.56596.Ctg.6192) was used as an internal reference gene (S8 File). One microgram of total RNA from each sample was treated with the PrimeScript RT reagent Kit with gDNA Eraser (Takara Bio, Japan) to remove any residual genomic DNA and convert it to cDNA, following the manufacturer protocol. The relative quantification was performed on Eco Real-Time PCR System (San Diego, California) using SYBR Premix Ex Taq (Takara Bio, Japan). The comparative 2^−ΔΔCt^ method [46] was used for relative quantification of the gene expression levels. The level of significance difference was calculated with independent-samples t-test (P<0.05).

## Results

### Field experimental, sequencing, de novo transcriptome assembly, and functional annotation

**Fig 2:**
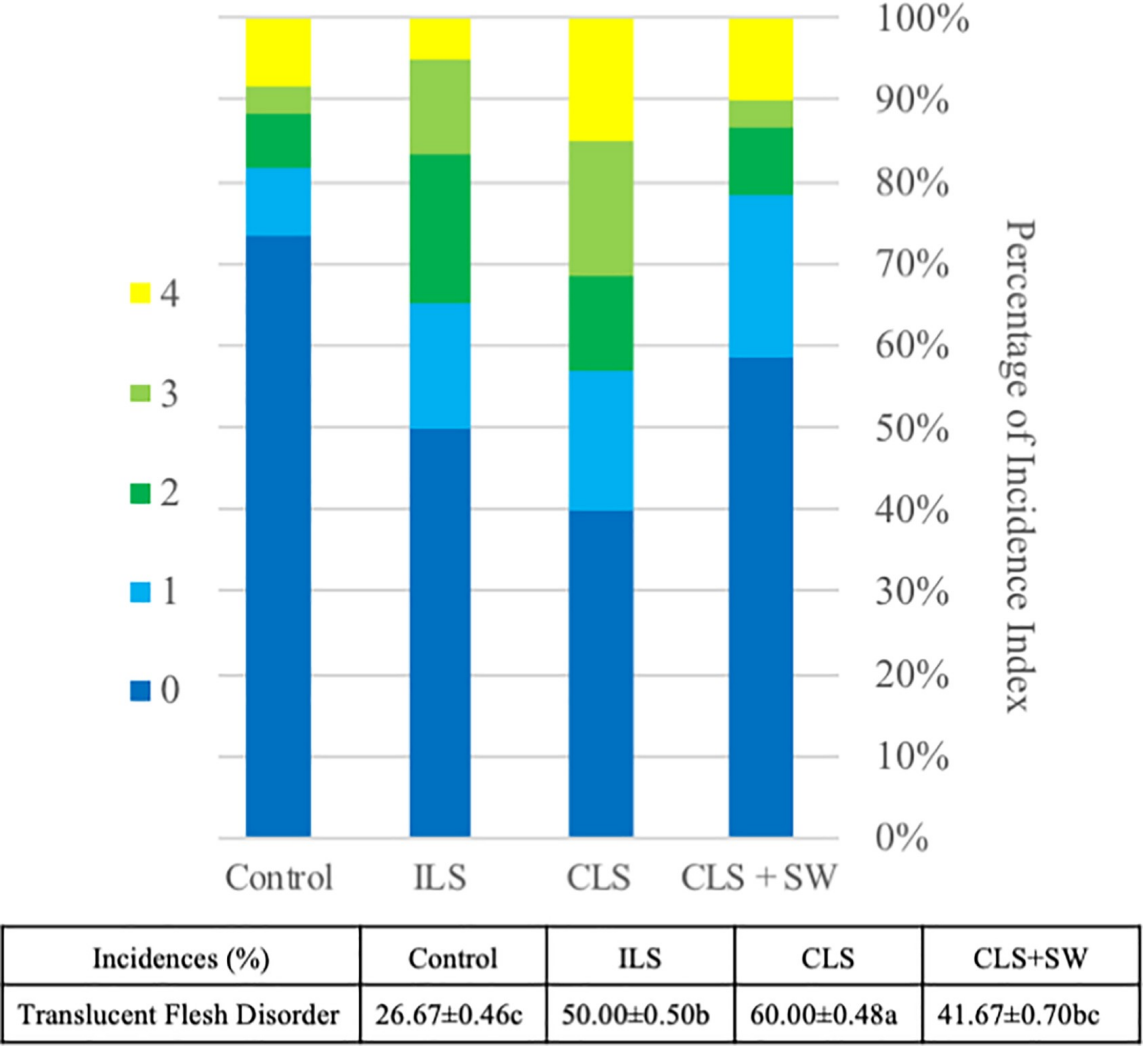
Incidences percentage of gamboge disorder and translucent flesh disorder in mangosteen during the treatment. (ILS: Irrigated-land Surface, CLS: Covering-land Surface, CLS + SW: Covering-land Surface + Supplied-water). The data for incidences is average from three trees as a replicate of each treatment. Data are represented as means±SD. The same letter within the same row are not significantly difference by Duncan’s Multiple Range.

The covered land surface (no irrigation) treatment showed incidence values of 60.00 ± 0.489% for TFD, higher than those of the other treatments (Fig 2). However, the incidence of total of physiological disorders was not significantly different between treatments. In the field experiment, TFD was scored [27]. The incidence of TFD was highest for the covered land surface (no irrigation), with an average score of 60%, and lowest for the control (26.67%). The scores for irrigated land surface and covered land surface with irrigation were 2 (18.33%) and 1 (20.00%), respectively.

An overview of the sequencing and *de novo* transcriptome assembly statistics is provided in Table 1. In total, 520,709,829 bp of clean reads (after removal of adaptors and low-quality reads) were obtained from 760,917,051 raw reads. The length of reads was between 35–230 bp and the average was 100-bp pooled-clean reads were assembled *de novo* using the Trinity program, resulting a total of 419,385 transcripts corresponding to 268,406 unigenes. The length of transcript and unigenes ranged from 201–12,603 bp. After removing redundancy and sorting, 183,274 contigs were produced with length ranging from 173–13,035 bp.

**Table 1:** Summary of reads and transcriptome assembly from mangosteen (*Garcinia mangostana* L.).

Features			
Total raw reads and bases (bp)	760,917,051 / 81,045,230,605		
Total processed reads and bases (bp)	520,709,829 / 51,955,852,570		
Read length (bp)	35–230		
Read mean (bp)	100		
	Trinity Output		Contig
	Transcripts	Unigenes	
Total Number	419,385	268,406	183,274
total assembled bases (bp)	184,084,221	104,000,468	90,822,801
Length range (bp)	201–12,603	201–12,603	173–13,035
N50 length (bp)	482	381	331
average (bp)	438.94	387.47	495.56

**Fig 3:**
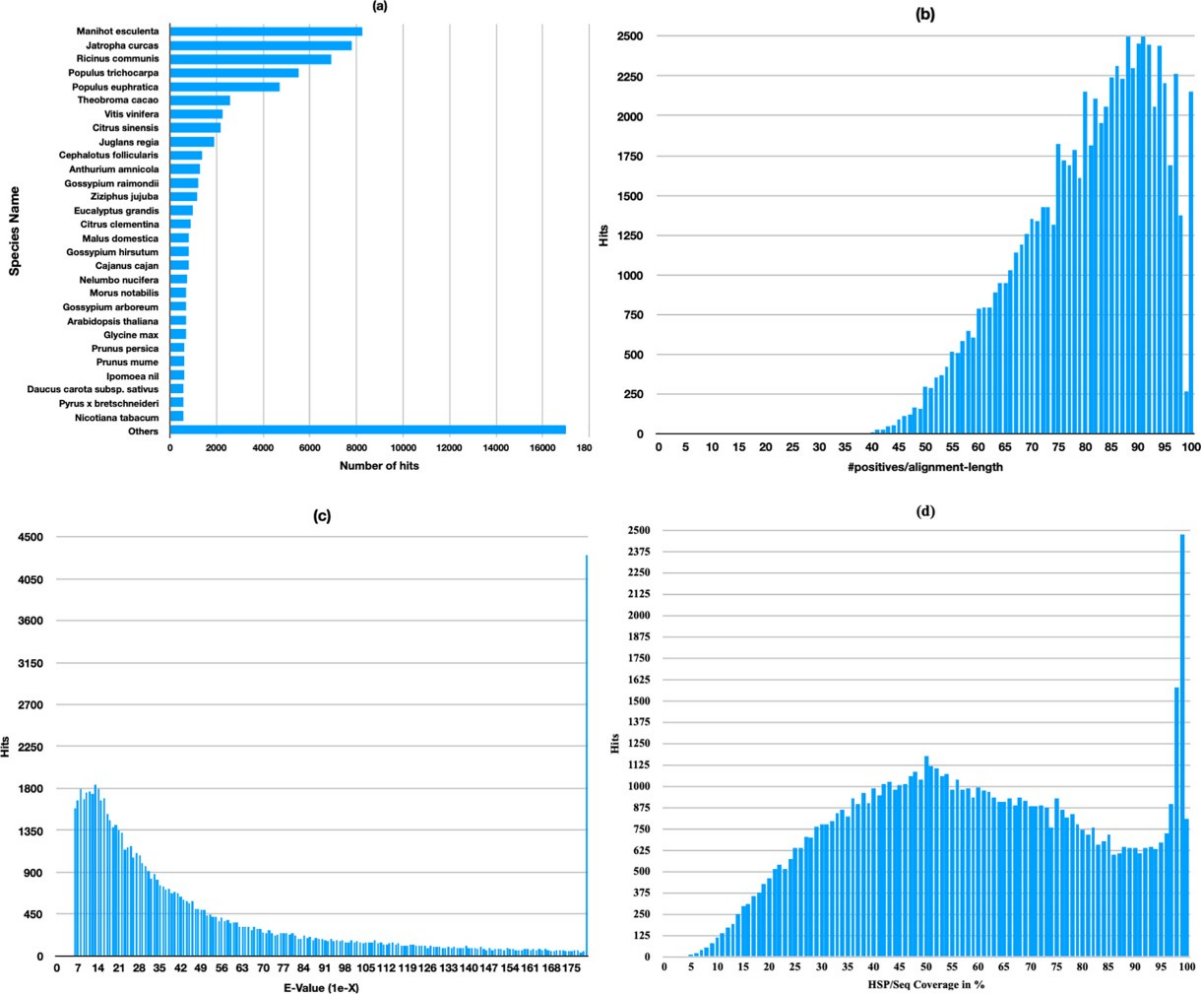
Homology search of *Garcinia mangostana* unigenes. (a) Species distribution of sequences; (b) Number hit distribution per sequence length (c) E-value distribution of the BLASTX hits against the nr protein NCBI database (subset to *Viridiplantae*) using an E-value cutoff of 10^−5^; (d) Similarity distribution of the BLASTX hits.

In total, 183,274 contigs were annotated and are presented in Table 2. Functional annotation was performed against several public databases. The database was subset into the Viridiplantae database to reduce false positive sequence homology from other organisms. The Contigs from the NCBI non-redundant (nr) protein database were used to analyze gene ontology, INTERPRO, and KEGG. An overview of species distribution, e-value distribution, number of hits, and alignment-length are presented in Fig 3. In total, 74,349 (40.57%) and 47,660 (26.00%) of contigs were tagged with the best aligning sequence homology from non-redundant proteins and nucleotides of NCBI databases, respectively. In comparison, 84,222 (45.95%) and 56,815 (31.00%) of contigs were annotated using UniProt KB and SwissProt databases, respectively.

**Table 2:** Number of sequences annotated by various databases.

No.	Database	Number of Sequences
1	Non redundant Protein NCBI*	74,349 (40.57%)
2	Non redundant Nucleotide NCBI*	47,660 (26.00%)
3	SwissProt*	56,815 (31.00%)
4	UniProt KB*	84,222 (45.95%)
5	InterPro**	73,567 (40.14%)
6	GO**	30,883 (8.76%)
7	KEGG**	2,504 (1.36%) with 138 pathways
8	TF/TR***	2,778 (1.52%)
9	PK***	1,673 (0.91%)

With asterisk, Analyzed by (*) BLAST+ program

(**) Blast2GO software, and

(***) iTAK program

**Fig 4:**
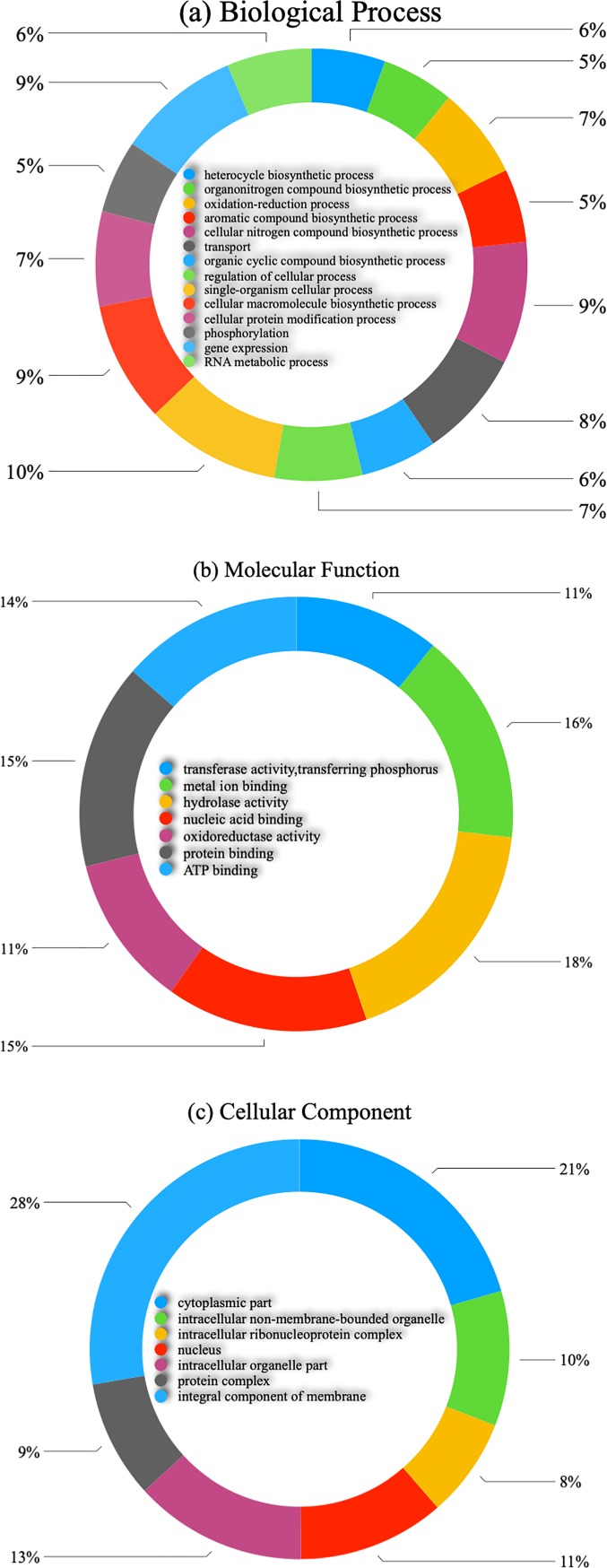
Gene Ontology (GO) classification of *G*. *mangostana*. (a) Biological Process; (b) Molecular Function; (c) Cellular Component. The numbers of each categories represent number of unigenes.

Gene Ontology (GO) terms and KEGG were retrieved from the Blast2GO database with associated BLAST search results using the NCBI non-redundant protein database. GO assigned contigs into three main GO categories, biological processes (BP), molecular function (MF), and cellular component (CC) (Fig 4). A total of 30,883 contigs had retrieved GO terms (S1 File). The highest abundance GO term from each category was identified. Hydrolase activity (2,225), metal ion binding (1,955), and protein binding (1,678) were the largest GO terms within the molecular function category. Single-organism cellular process (2,081), gene expression (1,896), and cellular nitrogen compound biosynthetic process (1,813) were the largest GO terms for biological process. Of the cellular component GO terms, integral component of membrane (2,448), cytoplasmic part (576), and intracellular organelle part (1,178) were the most represented. KEGG biological pathway analysis showed that 2,504 contigs were mapped into 138 KEGG pathways (S2 File). Purine metabolism pathway was the largest pathway assigned to the contigs (1,008), followed by thiamine metabolism (542), biosynthesis of antibiotics (476), and aminobenzoate degradation (257), respectively.

In total, 2,778 contigs were assigned to 64 families of putative transcription factors (TFs) and 25 families of transcriptional regulators (TRs), as well as 1,673 assigned to 117 families of protein kinases (PKs) (S3 File). Three transcription factors, WRKY (188 TFs), C3H Zinc finger (165 TFs), and MYB-related (152 TFs), respectively, were the most annotated transcription factors. Three transcriptional regulators, AUX/IAA (88 TRs), SNF2 (52 TRs), and mTERF (51 TRs), were the most annotated transcriptional regulators.

### Differentially expressed genes (DEGs) and their functional annotation analyses

**Fig 5:**
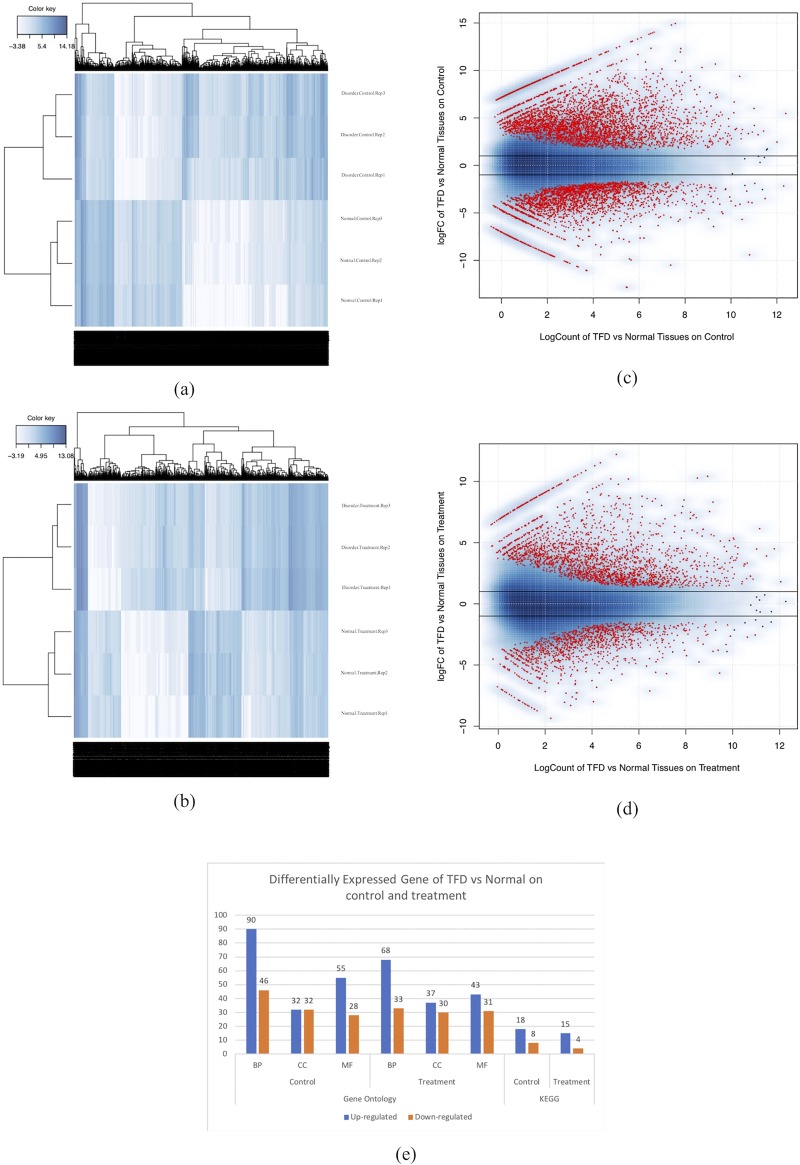
Overview of DEGs between pairwise comparison. DEG hierarchal clustering of normal aril versus disorder-affected aril on (a) control condition and (b) treatment condition. DEG Heatmap of up-regulated and down-regulated on normal aril versus disorder-affected aril on (c) control condition and (d) treatment condition. (e) Distribution Number of GO and KEGG annotation for DEG normal aril versus disorder-affected aril on control condition and treatment condition.

Using the Bowtie2 program, clean reads from 12 libraries were mapped individually onto the mangosteen transcriptome as a reference. Finally, count data were performed by the eXpress program before differential expression analysis using the edgeR program. For global DEGs analysis, counted-reads from 12 libraries were subjected to two pairwise comparisons, namely TFD vs normal aril in both control (unstressed water) and treatment (under water deficit) conditions. In total, 51,239 contigs for the control and 46,564 contigs for the treatment condition were used for DEG analysis after a reasonable level filtering from each sample and differences between samples were shown by hierarchal clustering analysis (Fig 5). DEGs were characterized after normalized-library by using TMM normalization and the normalized expression level using count per million (CPM). The number of DEGs was identified from each pairwise comparison. A cutoff was applied with FDR threshold of 0.01, after which 6228 DEGs under the control condition and 3327 DEGs in treatment condition (under water deficit condition) remained (S4 File).

The up-regulated and down-regulated DEGs were identified (Fig 5). The top ten up-regulated and down-regulated DEGs from each pairwise comparison is shown in Table 3 and all DEGs are listed in S4 File. Functional annotations of DEG for GO terms and KEGG pathway (Table 4 and S5 File) were annotated by using SwissProt accession on the David 6.8 and KOBAS 2.0 web interfaces.

**Table 3:** Top ten up-regulated and down-regulated of DEGs from each pairwise comparison.

Contig Name	SwissProt ID	Gene Description	logFC	logCPM	PValue	FDR
Control Condition						
C12506.97321.Ctg.24200	Q9SV43	Patatin-like protein 7	14.95	7.57	3.57E-13	1.89E-10
C12506.8678.T85127.c3.g1.i2	Q9FZ62	Inorganic pyrophosphatase 2	14.79	7.41	1.65E-11	4.69E-09
C12506.10653.Ctg.6487	Q9FE06	Protein EXORDIUM-like 2	14.03	6.65	3.26E-17	6.18E-14
C12506.70689.T89816.c0.g4.i2	O24248	Major allergen Pru av 1	13.95	6.57	9.04E-09	7.53E-07
C12506.10652.Ctg.6486	Q9FE06	Protein EXORDIUM-like 2	13.88	6.50	6.94E-18	1.69E-14
C12506.8673.Ctg.214	Q9FZ62	Inorganic pyrophosphatase 2	13.81	6.42	1.12E-10	2.20E-08
C12506.13345.Ctg.2938	O04887	Pectinesterase 2	13.47	6.09	3.01E-15	2.66E-12
C12506.94102.Ctg.27348	P13917	Basic 7S globulin	13.31	5.93	1.69E-14	1.20E-11
C12506.106460.Ctg.14073	P92519	Uncharacterized mitochondrial protein AtMg00810	13.08	5.70	1.68E-15	1.53E-12
C12506.2850.Ctg.17387	Q9FU27	Zinc finger CCCH domain-containing protein 2	12.52	5.14	5.41E-22	4.62E-18
C12506.81311.T85281.c0.g1.i1	F4JZL7	Heavy metal-associated isoprenylated plant protein 33	-9.45	2.14	1.65E-09	1.90E-07
C12506.61978.T97705.c0.g1.i1	P0C2F6	Putative ribonuclease H protein At1g65750	-9.48	2.17	1.52E-08	1.12E-06
C12506.48406.Ctg.30552	Q9FLX5	ABC transporter G family member 8	-9.48	2.18	5.70E-10	7.98E-08
C12506.79752.Ctg.35759	Q5BPJ0	Protein trichome birefringence-like 11	-9.50	2.19	2.21E-11	6.09E-09
C12506.5600.Ctg.45566	Q9FME8	Oligopeptide transporter 4	-9.52	2.21	2.96E-09	3.09E-07
C12506.53477.T71605.c0.g1.i1	Q9SZL7	Protein FAR1-RELATED SEQUENCE 9	-9.56	2.25	2.42E-08	1.62E-06
C12506.52536.T93669.c3.g1.i5	Q93ZF5	Phosphate transporter PHO1 homolog 1	-9.60	2.28	2.03E-10	3.59E-08
C12506.52536.T93669.c3.g1.i3	Q93ZF5	Phosphate transporter PHO1 homolog 1	-9.63	2.32	9.02E-10	1.16E-07
C12506.52532.T58363.c0.g1.i1	P48979	Polygalacturonase	-10.13	2.80	9.36E-14	5.64E-11
C12506.52521.Ctg.42538	Q40392	TMV resistance protein N	-10.50	7.37	1.23E-19	5.74E-16
Treatment Condition						
C12506.4356.T83669.c1.g5.i1	Q9LDM4	Cyclin-B2-3	12.21	5.04	2.75E-19	2.13E-16
C12506.7409.Ctg.12905	Q9LK35	Receptor-like protein kinase THESEUS 1	11.28	4.12	4.24E-25	8.23E-22
C12506.19976.T95192.c0.g7.i2	Q9ZSA7	Protein DMR6-LIKE OXYGENASE 2	10.43	8.93	1.23E-28	5.75E-25
C12506.13345.Ctg.2938	O04887	Pectinesterase 2	10.38	3.23	1.19E-16	5.54E-14
C12506.14846.Ctg.28664	P22195	Cationic peroxidase 1	10.28	8.49	2.48E-26	6.09E-23
C12506.4354.T83669.c1.g1.i1	Q0D9C7	Cyclin-B2-2	10.21	4.80	4.69E-16	1.95E-13
C12506.43836.T86003.c0.g3.i1	Q95661	Small heat shock protein, chloroplastic	10.20	5.55	1.17E-19	9.78E-17
C12506.26827.T86003.c0.g1.i1	Q95661	Small heat shock protein, chloroplastic	10.13	2.98	4.88E-10	6.17E-08
C12506.61319.T64003.c0.g1.i1	Q9FU27	Zinc finger CCCH domain-containing protein 2	10.08	2.93	6.06E-17	2.97E-14
C12506.60469.T62745.c0.g1.i1	O22893	Galactinol synthase 1	9.79	2.65	1.13E-12	2.54E-10
C12506.29228.T79437.c0.g1.i1	O81701	Polcalcin Aln g 4	-6.87	1.73	8.96E-09	8.24E-07
C12506.25153.T83650.c0.g2.i3	F4IFN6	DNA polymerase epsilon catalytic subunit B	-6.89	1.73	4.47E-07	2.43E-05
C12506.23037.T85364.c1.g1.i1	P93313	NADH-ubiquinone oxidoreductase chain 4	-7.12	6.46	1.98E-04	3.75E-03
C12506.50164.T93754.c1.g7.i3	Q9FH21	Protein DETOXIFICATION 55	-7.29	3.36	2.16E-13	5.45E-11
C12506.5887.T60388.c0.g1.i1	Q0WPX7	BAG family molecular chaperone regulator 2	-7.40	0.48	9.13E-07	4.44E-05
C12506.58875.T21775.c0.g1.i1	P33154	Pathogenesis-related protein 1	-7.51	0.56	1.02E-06	4.87E-05
C12506.46627.T96113.c9.g1.i2	Q9SCW1	Beta-galactosidase 1	-8.00	4.06	6.68E-11	1.02E-08
C12506.97059.Ctg.37751	Q94KL8	Secoisolariciresinol dehydrogenase (Fragment)	-8.30	3.09	1.29E-09	1.45E-07
C35962.0.T79648.c0.g1.i1	Q9LYC8	Monothiol glutaredoxin-S6	-8.30	1.25	5.04E-07	2.67E-05
C12506.59705.T88136.c0.g1.i3	B5WWZ8	Long-chain-alcohol oxidase FAO1	-8.61	1.55	2.57E-07	1.50E-05

**Table 4:** Top three significantly GO functional annotation categories of DEGs from each pairwise comparison.

Category	Term	%	PValue	FDR
Control condition				
Up-regulated				
BP	GO:0006355~regulation of transcription, DNA-templated	12.77	2.22E-06	3.51E-03
BP	GO:0006351~transcription, DNA-templated	12.40	2.48E-08	3.92E-05
BP	GO:0006952~defense response	5.00	1.74E-05	2.75E-02
CC	GO:0005634~nucleus	37.74	4.90E-06	6.23E-03
CC	GO:0005737~cytoplasm	21.18	1.72E-10	2.18E-07
CC	GO:0005886~plasma membrane	17.58	2.99E-08	3.80E-05
MF	GO:0005524~ATP binding	15.36	3.81E-10	5.65E-07
MF	GO:0005515~protein binding	13.32	3.71E-11	5.51E-08
MF	GO:0003677~DNA binding	10.82	1.86E-03	2.73E+00
Down-regulated				
BP	GO:0006468~protein phosphorylation	4.59	6.80E-02	6.62E+01
BP	GO:0015031~protein transport	4.08	8.20E-08	1.26E-04
BP	GO:0006810~transport	2.93	1.37E-03	2.09E+00
CC	GO:0016021~integral component of membrane	24.62	4.67E-07	5.95E-04
CC	GO:0005886~plasma membrane	20.79	1.28E-12	1.63E-09
CC	GO:0009507~chloroplast	16.71	4.52E-04	5.75E-01
MF	GO:0005515~protein binding	12.24	1.21E-06	1.74E-03
MF	GO:0005524~ATP binding	11.99	7.65E-03	1.04E+01
MF	GO:0046872~metal ion binding	9.44	4.54E-04	6.48E-01
Treatment Condition				
Up-regulated				
BP	GO:0006351~transcription, DNA-templated	9.24	5.46E-02	5.73E+01
BP	GO:0009651~response to salt stress	5.16	1.08E-07	1.64E-04
BP	GO:0009737~response to abscisic acid	4.35	6.43E-07	9.75E-04
CC	GO:0005634~nucleus	36.68	2.09E-04	2.59E-01
CC	GO:0005737~cytoplasm	22.55	5.29E-11	6.55E-08
CC	GO:0005829~cytosol	17.66	1.11E-21	1.38E-18
MF	GO:0005515~protein binding	14.81	3.16E-12	4.46E-09
MF	GO:0005524~ATP binding	14.27	6.90E-06	9.76E-03
MF	GO:0046872~metal ion binding	8.56	1.13E-02	1.48E+01
Down-regulated				
BP	GO:0006468~protein phosphorylation	5.82	1.95E-02	2.53E+01
BP	GO:0006810~transport	3.80	1.33E-03	1.95E+00
BP	GO:0009651~response to salt stress	3.54	4.34E-02	4.81E+01
CC	GO:0016021~integral component of membrane	29.87	1.16E-09	1.37E-06
CC	GO:0005886~plasma membrane	26.84	4.07E-16	5.22E-13
CC	GO:0016020~membrane	10.89	1.25E-04	1.47E-01
MF	GO:0005524~ATP binding	13.92	2.29E-03	3.07E+00
MF	GO:0005515~protein binding	9.62	8.80E-02	7.14E+01
MF	GO:0046872~metal ion binding	8.61	5.89E-02	5.62E+01

BP: Biological Processes, CC: Cellular Component, MF: Molecular Function

Under the control condition, a total of 6,228 DEGs was identified. 2162 DEGs were up-regulated and 4066 DEGs were down-regulated. The highest up-regulated of DEGs involved Patatin-like protein 7, Inorganic pyrophosphatase 2, Protein EXORDIUM-like 2, etc., and the highest down-regulated of DEGs involved Phosphate transporter PHO1 homolog 1, Polygalacturonase, TMV resistance protein N, etc. GO terms assigned 136 terms of biological process, 64 terms of cellular component, and 83 terms of molecular function, respectively. The top 3 most counted enriched GO functional annotation categories were GO:0006355 (regulation of transcription, DNA-templated), GO:0006351 (transcription, DNA-templated), and GO:0006952 (defense response) for the biological process category, GO:0005634 (nucleus), GO:0016021 (integral component of membrane), GO:0005737 (cytoplasm) for the cellular component category, and GO:0005524 (ATP binding), GO:0005515 (protein binding), and GO:0003677 (DNA binding) for the molecular function category. DEGs were annotated against the KEGG pathways database and assigned 26 pathways involving ath01130 (biosynthesis of antibiotics), ath01230 (biosynthesis of amino acids), and ath01200 (carbon metabolism). Interestingly, carbohydrate metabolisms-related pathways such as glycolysis/gluconeogenesis, starch and sucrose metabolism, and pyruvate metabolism were highly observed.

Under the treatment condition, a total of 3,326 DEGs was identified. 2,162 DEGs were up-regulated and 1,164 DEGs were down-regulated. The highest up-regulated DEGs involved Cyclin-B2-3, Receptor-like protein kinase THESEUS 1, Protein DMR6-LIKE OXYGENASE 2, and pectinesterase 2. The most down-regulated DEGs included beta-galactosidase 1, secoisolariciresinol dehydrogenase, monothiol glutaredoxin-S6, and long-chain-alcohol oxidase FAO1. GO analysis assigned 101 terms of biological process, 67 terms of cellular component, and 74 terms of molecular function. The top 3 most counted enriched GO functional annotation categories were GO:0006351 (transcription, DNA-templated), GO:0006468 (protein phosphorylation), and GO:0009651 (response to salt stress) for the biological process category, GO:0005634 (nucleus), GO:0016021 (integral component of membrane), and GO:0005886 (plasma membrane) for the cellular component category, and GO:0005515 (protein binding), GO:0005524 (ATP binding, and GO:0046872 (metal ion binding) for the molecular function category. DEGs were annotated against the KEGG pathways database and assigned to 19 pathways involving ath01230 (biosynthesis of amino acids), and ath01200 (carbon metabolism).

### Identification of candidate genes putatively involved in occurrences of TFD-like physiological disorders

**Fig 6:**
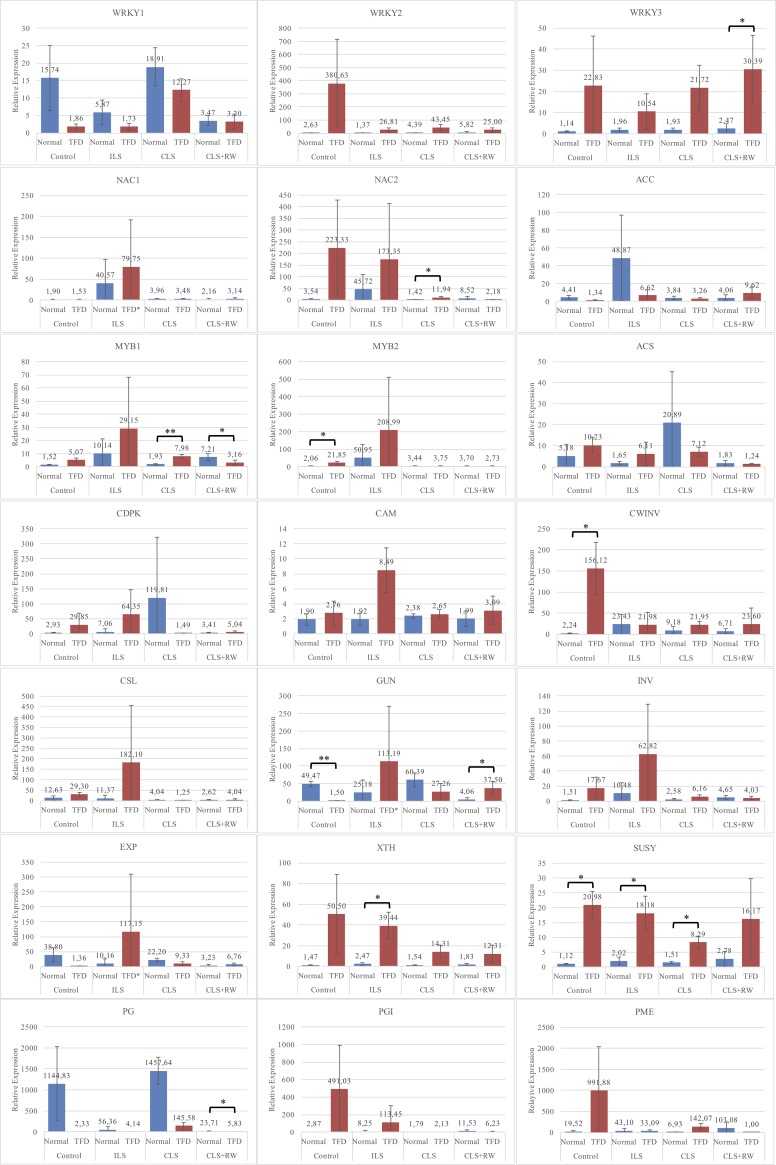
Validation for twenty-one selected genes of quantitative reverse transcription PCR (qRTPCR). The result was calculated using 2^-ΔΔCt^ method and the mean was three biological replicates (±SD). *Represents the level of significance difference P < 0.05, ** represents the level of significance difference P < 0.01 in independent-samples t-test.(1) control as natural growth condition (Control), (2) irrigating land surface under canopy with drip irrigation at whole day (ILS), (3) covering land surface under canopy with dark plastic (CLS), and (4) the condition as same as third conditions, but suddenly applied an irrigation every morning during one week from 9 weeks after anthesis (CLS+RW). ACO (1-aminocyclopropane-1-carboxylate oxidase), ACS (1-aminocyclopropane-1-carboxylate synthase), CAM (Calmodulin), CDPK (Calcium-dependent protein kinase), CINV (Alkaline/neutral invertase), CSL (Cellulose synthase-like protein), CWINV (Beta-fructofuranosidase), EXP (Expansin), GUN (Endoglucanase), MYB1 (Transcription factor MYB1R1), MYB2 (Transcription factor MYB44), NAC1 (Transcription factor NAC47), NAC2 (Transcription factor NAC29), PG (Polygalacturonase), PGI (Polygalacturonase inhibitor), PME (Pectinesterase), SUSY (Sucrose synthase), WRKY 1 (Transcription factor WRKY75), WRKY 2 (Transcription factor WRKY40), WRKY 3 (Transcription factor WRKY40), XTH (Xyloglucan endotransglucosylase/hydrolase).

We used GO-slim terms (S6 File) and KEGG pathway (S7 File) results annotated by using SwissProt accession on the KOBAS 2.0 web interface to determine related gene into mineral nutrition-related, phytohormone-related, maturation and ripening process-related, including cell wall modification and carbohydrate metabolism, and water-related genes. In total, 21 genes were selected for validity testing from the genes identified from contigs (Fig 6; S8 File). The candidate gene is also associated with several TFD-causing hypotheses, including genes associated with the ripening process. Genes involved in the process of cell wall synthesis and degradation became of interest to this study.

The polygalacturonase gene under TFD conditions showed decreased expression level compared to control conditions in all treatments, and this is noticeably different from the expression level of the gene polygalacturonase inhibitor, which has increased expression level under TFD conditions, compared to the control in all treatments except CLS+RW treatments with significant based statistical test. The endoglucanase (GUN) gene under TFD conditions showed decreased expression level compared in control conditions (statistically significant) and CLS treatment, however increased the expression level in ILS and CLS+RW (statistically significant) treatments. The sucrose synthase (SUSY) gene expression level showed a significant increase in TFD compared to normal conditions in almost treatments.

The PME gene showed very high expression level under TFD conditions compared to normal under control and CLS conditions, compared to the stable condition of ILS but in the CLS+RW condition, its expression level showed lower expression in TFD than normal tissue. The XTH gene showed an increased pattern of expression level in TFD compared to normal samples under all treatment conditions and only in ILS treatment showed statistically significant. The EXP gene increased in expression level in the TFD compared to normal samples under control and CLS conditions, but was inversely proportional under both other conditions. Invertase genes in the cell wall and cytoplasm tended to have increased expression level under TFD conditions compared to controls in almost all treatments.

ACC gene expression showed decreased expression level in TFD compared to normal samples in all treatments except CLS+RW. While the ACS gene showed an increase in TFD compared to controls in the control treatment and ILS, the conditions were reversed under CLS and CLS+RW conditions. Calcium-related genes, such as CAM, had increased expression level in TFD compared to controls under all treatments. However, CDPK gene expression level exhibited the same pattern except under CLS conditions that had decreased expression level under TFD conditions than controls.

Three types of transcription, i.e. NAC, MYB, and WRKY, have been identified to be related to the three major transcription factors. MYB1 and MYB2 show increased expression level in TFD tissue compared to normal tissue in almost all treatments except in CLS+RW treatments. NAC1 did not show significant differences between TFD and normal tissue, except for under the ILS treatment. NAC2 showed significant differences between TFD and normal tissue under normal and ILS conditions, whereas under CLS and CLS+RW conditions, it did not show significant differences. In contrast, WRKY1 showed higher expression level than TFD tissue under almost all treatments. WRKY2 and WRKY3 showed higher expression level in TFD than normal tissue under almost all treatments. WRKY2 shows significant expression level under normal treatment conditions. WKRY3 showed significant differences in expression level between TFD and normal tissues.

## Discussion

The present study is to report transcriptomic analysis for elucidating the physiological disorders in mangosteen, namely translucent flesh disorder responses to different water regimes. Due to the lack of genomic and transcriptomic data for mangosteen, we used a massive sequencing technology called RNA Sequencing (RNA-seq) to read whole transcripts and determine their gene expression level. In the present study, we analyzed approximately 700 million raw reads (80-gb bases) and 183,274 contigs were generated by de novo assembly using the Trinity program after removal of redundant contigs. More than 50% of the contigs were annotated by at least one public database, such as the non-redundant (nr) protein of NCBI database and SwissProt of UniProtKB databases.

In apple, approximately 18 factors have been identified as either causing or being correlated with watercore such as water regime, temperature, mineral nutrition, source/sink ratio, and maturation or ripening processes [6]. Environmental factors such as high temperature [47] and sunlight exposure [48] have also been found to be highly correlated to increased incidence of watercore in Japanese pear. Physiological and biochemical changes also determined and were correlated with the incidence of TFD-like physiological disorders. Alteration of carbohydrate metabolism may play a role in increasing the incidence of watercore in apple [49], flesh translucency in pineapple [11], and water-soaked flesh disorder in peach [9]. A particular calmodulin-binding protein (CaM-BP) has also been observed to be absent in water-soaked melon fruit [10].

The results of this study indicate that the sucrose synthase (SUSY), endoglucanase (GUN) and polygalacturonase (PG) genes involved in the incidence of TFD in mangosteen are similar to those in other plants, such as watercore incidence in pear and apple. An increase in gene expression level associated with ripening such as sugar metabolism, cell wall loosening, and degradation related genes was observed. In apple, sorbitol transporter (mdSOT) became one of the indicators of watercore, due to an increase in expression level [50]. Sucrose synthase (SUSY) also provides an indicator accumulation of sucrose [51].

TFD tissue had a thickened cell wall was thicker and showed the disintegration of the middle lamella [24]. This cell state indicates the occurrence of cell wall loosening and disintegration, which allows strong cell wall strength to become weak and ultimately damaged. Cell walls are composed of carbohydrate complexes in the form of polysaccharides, such as cellulose and pectin. The presence of middle lamella disintegration may allow formation of pectin by the de-esterification homogalacturonan complex [52], as seen from the increased pectin methylesterase (PME) expression level compared to normal tissue. This can also be attributed to to the deficiency of calcium, thereby crosslinking between the pectin in the cell tissue is not formed. Conversely, high expression of pectin methylesterases (PMEs) increases Ca^2+^ bound to the cell wall [53]. Thus, the application of calcium can reduce the incidence of TFD [21].

Previous studies have shown that the availability of water affects the incidence of TFD. Availability of water is important for plants; fluctuation in water levels leads to instability in plant potential and caused the fragile cell wall to rupture [54]. Irrigation treatment on a daily basis induced the incidence of TFD; that explained it as loss of turgor pressure due to the accumulation of apoplastic solutes [55]. Continuous water application increases the incidence of TFD compared with other treatments [19]. The incidence of TFD was different among treatments. For both the irrigated land surface and covered land surface, the incidence of TFD was higher than in the other conditions. Similarly, TFD incidence was increased by daily watering or interval watering (7- or 4-day intervals) relative to the control (no irrigation added) [23]. The covered land surface with irrigation had a lower incidence of TFD than the irrigated land surface and covered land surface by 8–9 weeks after anthesis, although it still had a higher incidence of TFD than the control. Nine weeks after anthesis was a time at which water supply had a strong impact on TFD and GD incidence in mangosteen fruit [22]. TFD may be triggered by a water potential imbalance in the cells that results from excess water content in the fruit, which itself arises from continuous exposure of the plants to high soil water potential during the pre-harvest period. Water potential gradients are also involved in water uptake by the roots and transpiration in the leaves [56].

The suppression of the pectate lyase mRNA in transgenic strawberry fruits during ripening resulted in firmer fruits [57]. On the other hand, the XTH gene shows an increase in TFD that allows expression level in the maintenance structure compared to cell wall disassembly that enlarges the cell wall (loosening). Cell size in TFD is significantly increased than in the normal aril [24]. This cell enlargement is likely due to the modified cell wall due to the loosening process. This is demonstrated in the expression level of genes associated with loosening, including expansin and XTH. EXP mediates cell wall loosening [58]. The polygalacturonase gene decreases in TFD compared to normal conditions as it acts in the softening process, resulting in degradation or solubilization of the cell wall. Increased pectinesterase expression level in TFD showed that the pectin is bound to the cell walls and pectin-bound calcium is higher than normal [25]. Calcium-pectate forms cross-links between homogalacturonan (HGA) in the middle of the lamella so that the pectin-bound calcium is increased.

**Fig 7:**
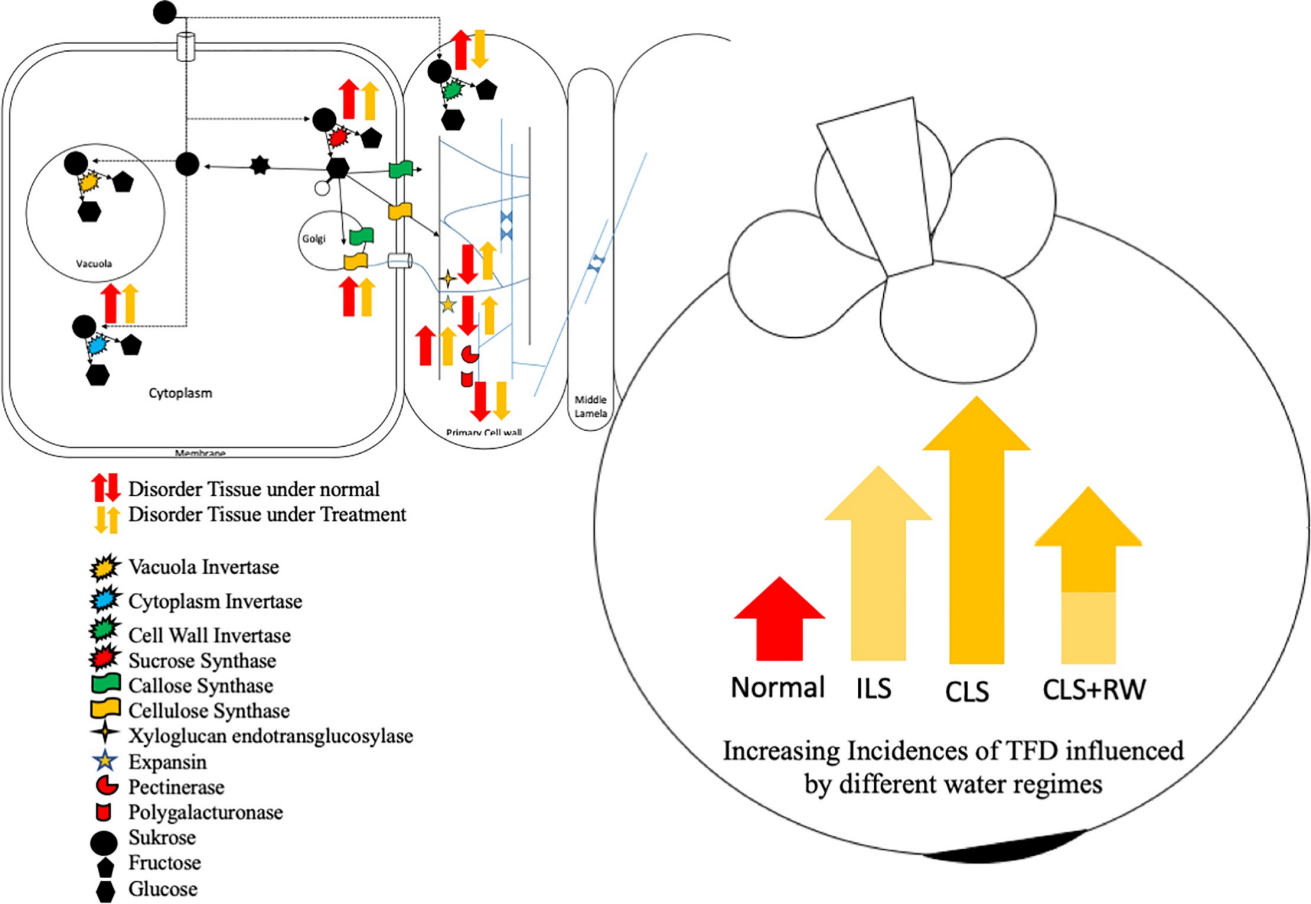
Model for up-regulated and down-regulated of genes related to TFD incidences under normal and treatment conditions.

These results provide a basis for future studies on the molecular mechanisms of the incidence of TFD in mangosteen since the mangosteen known as polyploid plant [59]. The present study summarized incidence of TFD by the following (Fig 7): first, the cells undergo major osmotic changes between the inside and outside of the cell so that cell membranes push against the cell wall due to mass migration into cells by osmosis (over-turgor pressure) [54]. Second, at the same time the cell wall responds by stretching the cell (loosening). This can be measured by CSL, XTH, and EXP gene expression level compared to normal tissue on the cell wall. In the middle lamella, PME gene expression level, which is the process of formation of the homogalacturonan complex, is also higher. In condition of calcium deficiencies, the calcium-pectate complex is not formed which causes the lamella damage. Third, because of the large flow of water into the cell, and the damaged middle lamella, water fills the air cavity. However, regarding physiological disorders, e.g. watercore in apple or pear, there are other factors that cause TFD occurrence, such as temperature changes, and hormone factors such as gibberellin. Thus, further studies are needed to re-confirm the genes-related translucent flesh disorder using RT-qPCR and also elucidate the other gene-related disorders such as gamboge disorder.

## Supporting information

S1 File Contigs annotation by Gene ontology and InterPro scan. (XLSX)

S2 File Contigs annotation by KEGG analysis. (XLSX)

S3 File Contigs annotation by Transcription factor, transcription regulator, and protein kinase. (XLSX)

S4 File Differentially expressed gene (DEG) of normal and TFD-affected aril from control and treatment conditions. (XLSX)

S5 File Annotations of differentially expressed gene (DEG) of normal and TFD-affected aril from control and treatment conditions by gene ontology and KEGG using David. (XLSX)

S6 File Annotations of differentially expressed gene (DEG) of normal and TFD-affected aril from control and treatment conditions by gene ontology using KOBAS. (XLSX)

S7 File Annotations of differentially expressed gene (DEG) of normal and TFD–affected aril from control and treatment conditions by KEGG using KOBAS. (XLSX)

S8 File List of reference genes for quantitative real time PCR analysis. (XLSX)
